# Effects of *Bifidobacterium longum* BB536 and *Bifidobacterium breve* MCC1274 on Body Composition in Normal and Overweight Adults in Randomized Placebo-Controlled Study

**DOI:** 10.3390/nu16060815

**Published:** 2024-03-13

**Authors:** Soichiro Sato, Satoshi Arai, Kumiko Kato, Keisuke Yoshida, Noriyuki Iwabuchi, Toru Sagami, Miyuki Tanaka

**Affiliations:** 1 R&D Division, Innovative Research Institute, Morinaga Milk Industry Co., Ltd., 5-1-83, Higashihara, Zama 252-8583, Japan; 2 Shinagawa Season Terrace Health Care Clinic, 1-2-70, Konan, Minato-ku 108-0075, Japan

**Keywords:** body composition, abdominal fat, serum triglycerides, probiotics

## Abstract

Visceral fat accumulation is considered to be associated with a higher risk of chronic diseases. We investigated the effects of *Bifidobacterium longum* subsp. *longum* (*B. longum*) BB536 and *Bifidobacterium breve* (*B. breve*) MCC1274 on body composition, including visceral fat, in a randomized, parallel-group, placebo-controlled study. Participants were between 29 and 64 years of age and had a body mass index (BMI) of greater than 23 and less than 30. One hundred participants were randomly assigned to the probiotics group or placebo group. Participants were administered probiotic capsules containing 1 × 10^10^ colony-forming units (CFUs) of *B. longum* BB536 and 5 × 10^9^ CFU of *B. breve* MCC1274 or placebo capsules without bifidobacteria for 16 weeks. In the probiotics group, abdominal visceral fat area, total abdominal fat area, and serum triglyceride levels were significantly decreased compared to those in the placebo group. Additionally, the increase in BMI observed in the placebo group was significantly suppressed in the probiotics group. This study showed that *B. longum* BB536 and *B. breve* MCC1274 reduced abdominal visceral fat and total fat levels in healthy normal and overweight adults, suggesting their beneficial effects on body composition.

## 1. Introduction

The prevalence of overweight and obesity is steadily increasing regardless of age, sex, ethnicity, etc. [1]. Obesity is known to be involved in the development of lifestyle diseases such as dyslipidemia and diabetes [2]. One of the characteristics of obesity is fat accumulation. Body fat is mainly classified into visceral and subcutaneous fat, and visceral fat accumulation is especially considered to be associated with a higher risk of chronic diseases [3]. The cause of obesity has been suggested to be related to genetic and environmental factors such as decreased physical activity and overeating [4]. Lifestyle modification, including calorie restriction and regular exercise, could be effective in ameliorating obesity, but it is difficult for individuals to maintain their lifestyle changes [5].

The intake of probiotics is expected to prevent and improve body composition [6]. Bifidobacteria are major probiotic bacteria, and according to a meta-analysis, they were particularly depleted in obese people [7]. The positive effects of bifidobacteria on serum lipid or body fat have been demonstrated in animal and clinical studies. Fermented milk containing *B. longum* BB536 was shown to reduce blood triglyceride levels elevated by a high-fat diet in vivo compared to those with fermented milk without *B. longum* BB536 [8]. *B. breve* MCC1274 was reported to enhance intestinal barrier function in vitro and to stimulate the expression of lipid metabolism genes in the liver in vivo [9,10]. Furthermore, intake of *B. breve* MCC1274 reduced body fat levels in normal and overweight adults [11,12,13]. In addition, the combination of *B. longum* BB536, *B. breve* MCC1274, and N-acetylglucosamine reduced BMI, body fat levels, and total abdominal fat area in healthy overweight participants [14]. The visceral fat area also significantly increased from baseline after 24 weeks of intake in the placebo group but not in the test food group. Thus, the beneficial effects of *B. longum* BB536 and *B. breve* MCC1274 have been reported, and these probiotics could be expected to be more effective in maintaining adequate body composition when combined. These studies could demonstrate the effectiveness in maintaining or promoting well-being for healthy adults. A previous study employed an intervention comprising a combination of these bacteria and a substrate, N-acetylglucosamine, so it is possible that the synergistic or complementary effects were observed due to the inclusion of the substrate [14]. In this study, to clarify the effects of the probiotics without any substrates, we investigated the effects of *B. longum* BB536 and *B. breve* MCC1274 on body composition, including visceral fat area, in healthy normal and overweight adults with BMIs greater than 23 and less than 30.

## 2. Materials and Methods

### 2.1. Study Design and Ethics

A randomized, parallel-group, placebo-controlled study was conducted at the Shinagawa Season Terrace Health Care Clinic in Tokyo, Japan, from August 2022 to December 2022. This study was registered in the UMIN-CTR (UMIN000047852) after approval was obtained from the Ethics Committee of Kobuna Orthopedic Clinic (approval number: MK-2204-01, approval date: 12 May 2022). Before the study was conducted, the purpose and procedures of the study and the rights of the participants were fully explained to the participants, and written consent was obtained. The study was conducted in accordance with the Declaration of Helsinki (Fortaleza, revised in 2013) and the Ethical Guidelines for Life Science and Medical Research Involving Human Subjects (Ministry of Education, Culture, Sports, Science and Technology, Ministry of Health, Labour and Welfare, and Ministry of Economy, Trade and Industry Notification No. 1, 2021).

### 2.2. Participants

Participants were included according to the inclusion and exclusion criteria. The inclusion criteria were between 20 and 64 years of age and a BMI of greater than 23 and less than 30. The exclusion criteria were as follows: (1) a history of serious diseases such as cerebrovascular, cardiac, hepatic, gastrointestinal, endocrine, or metabolic diseases; (2) use of drugs that may affect obesity, hyperlipidemia, or lipid metabolism; (3) inability to stop consuming health foods or supplements that may affect obesity, hyperlipidemia, or lipid metabolism; (4) heavy smoking; (5) heavy drinking; (6) serious drug or food allergies; (7) metal implants at the computed tomography (CT) scan site due to surgery; (8) a cardiac pacemaker or implantable cardioverter-defibrillator; (9) pregnancy, lactation, or expectation of becoming pregnant during this study; (10) participating or intending to participate in other food studies, drug use studies, or cosmetic or drug application studies during this study; and (11) any other reason making participation inappropriate as deemed by the principal investigator.

### 2.3. Intervention

The test foods were probiotic capsules containing 1 × 10^10^ colony-forming units (CFUs) of *B. longum* BB536 and 5 × 10^9^ CFU of *B. breve* MCC1274 (B-3) per 2 capsules and placebo capsules without bifidobacteria. These test foods were provided by Morinaga Milk Industry (Tokyo, Japan). The taste, smell, and appearance of the test foods were confirmed to be indistinguishable by an independent investigator before and at the end of this study. Participants took 2 capsules with water daily for 16 weeks.

The intake of supplements including *Lactobacillus*, *Bifidobacterium*, or oligosaccharides was prohibited during this study. Participants were informed to refrain from consuming functional foods claiming fat reduction or improvement of obesity. If they ingested these prohibited things, they were recorded in the participant’s diary during the study period.

### 2.4. Primary Outcomes

The primary outcome was body composition. Abdominal fat was measured using CT with high accuracy (Supria Advance; Hitachi, Ltd., Tokyo, Japan). Visceral fat area, subcutaneous fat area, and total fat area were calculated using a software (FAT Scan Ver. 4.0; East Japan Technical Laboratory Inc., Ibaraki, Japan). In addition, body fat percentage and body fat mass were measured using a multifrequency bioelectrical impedance device (InBody 470; InBody Japan, Inc., Tokyo, Japan). All measurements were performed after an overnight fast and at the same time in the morning.

### 2.5. Secondary Outcomes

Secondary outcomes included the following items: anthropometric data (weight, BMI, waist circumference, waist–hip circumference ratio) and blood parameters (serum lipids, blood glucose-related variables, liver function, and inflammation markers).

Weight was measured using an Inbody 470, and BMI was calculated automatically using a manually measured value for height. Waist circumference was measured at the midpoint between the lower edge of the ribs and the upper edge of the iliac crest.

Blood samples were collected and analyzed by LSI Medience Corporation (Tokyo, Japan). Blood was collected after an overnight fast. Total cholesterol, low-density lipoprotein cholesterol, high-density lipoprotein cholesterol, triglycerides (TGs), fasting blood glucose, HbA1c, insulin, glycoalbumin, high-sensitivity C-reactive protein (hCRP), white blood cells (WBCs), red blood cells (RBCs), hemoglobin, hematocrit, platelets, leukocytes, total protein, albumin, total bilirubin, aspartate transaminase, alanine aminotransaminase, alkaline phosphatase, γ-glutamyl transpeptidase, lactate dehydrogenase, urea nitrogen, creatinine, uric acid, sodium, chloride, potassium, and calcium were measured via blood tests for efficacy and safety analysis.

All measurements were performed 4 weeks before the start of the intake period (baseline) and at the endpoint of the intake period (week 16).

### 2.6. Diary Survey

Participants recorded their intake of test foods, medications, physical condition, exercise, and diet in a diary. These records were kept daily from baseline until the endpoint of week 16. In addition, they recorded their diet and the quantity in detail for 3 consecutive days before the start of the intake period (week 0) and the endpoint of week 16. Dietitians analyzed the dietary records and calculated the 3-day average for total energy, protein, fat, carbohydrate, and fiber intake using Excel Eiyokun ver. 8.0 (Kenpakusha Inc., Tokyo, Japan).

### 2.7. Safety Assessment

At baseline and week 16, the principal investigator interviewed the participants to assess their health status. Blood and urine tests were performed at baseline and week 16. For the urine tests, urine protein, sugar, bilirubin, ketone bodies, occult blood, urobilinogen, pH, and specific gravity were measured. Adverse events were reported by the participants in their diaries or identified by blood and urine test results, judged according to the criteria of “Judgment criteria for adverse drug reactions and abnormal laboratory values in clinical trials with antimicrobial agents” by the Japanese Society of Chemotherapy. The severity of adverse events and the association between adverse events and the intake of test foods were evaluated by the principal investigator. The number and incidence of adverse events were calculated for each group.

### 2.8. Sample Size

The sample size was calculated with reference to a previous clinical study that showed a reduction in visceral fat area with probiotic intervention [15]. Based on the data on the change in visceral fat area (calculation of effect size 0.547), the needed number of participants was 86 using G*power 3.1.9.7 (http://www.gpower.hhu.de/, accessed on 31 January 2022) to detect a difference between groups at a 5% significance level with 80% statistical power. A total of 100 participants were required, considering a dropout rate of about 10%.

### 2.9. Randomization

Participants were randomly divided 1:1 into two groups using stratified randomization by an independent investigator. The stratification factor was visceral fat area (<100 and ≥100). The allocation sequence was sealed to both participants and investigators until the end of the study, and the database was locked.

### 2.10. Statistical Analysis

The primary population for the efficacy analysis was the per-protocol set (PPS) population. Baseline characteristics were compared by Fisher’s exact test for categorical variables and two-sample *t* test for continuous variables. Primary and secondary outcomes were analyzed by covariance (ANCOVA) adjusted for baseline values. In addition, within-group changes from baseline to week 16 were analyzed by a paired *t* test. The incidence of adverse events was compared between groups by Fischer’s exact test. Two-sided *p* values less than 0.05 were considered to indicate statistical significance. All statistical analyses were performed using IBM^®^ SPSS ver. 28.0 (IBM Corp, Armonk, NY, USA).

## 3. Results

### 3.1. Participants

**Figure 1:**
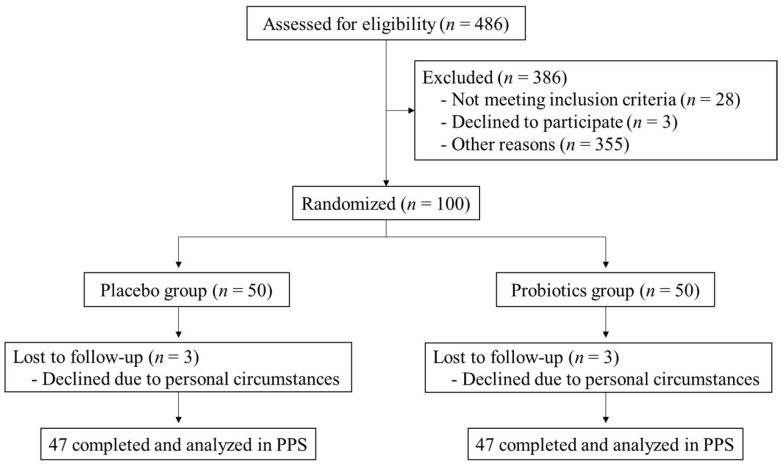
Study flow chart.

The study flow chart is shown in Figure 1. During recruitment (June 2022–July 2022), 486 participants provided written consent, and 386 participants were judged to be ineligible due to the failure to meet inclusion criteria (*n* = 28), declining to participate (*n* = 3), or other reasons (their medical history, medication status or the levels of blood parameters to exclude sick participants judged by the principal investigator, heavy drinking, serious allergy, etc.; *n* = 355). A total of 100 participants were enrolled according to the inclusion and exclusion criteria (placebo group; *n* = 50, probiotics group; *n* = 50). All participants were judged to be healthy based on interviews and blood and urine tests performed by the principal investigator. The safety analysis set (SAF) included 100 participants who consumed the test food at least once. In total, 6 participants (placebo group; *n* = 3, probiotics group; *n* = 3) dropped out during the intake period, and 94 participants completed the study. Thus, 94 participants (placebo group; *n* = 47, probiotics group; *n* = 47) were included in the PPS analysis.

### 3.2. Participant Characteristics

The baseline characteristics of the participants are shown in Table 1. All participants were between the ages of 29 and 64 (mean ± SD in placebo group; 50.11 ± 7.21, probiotics group; 50.43 ± 7.59). There were no significant differences in baseline information between the two groups. The nutritional composition at week 0 and week 16 was assessed for total energy, protein, fat, carbohydrate, and fiber intake (Table 2). For all nutrients, there were no items whose intake changed significantly from week 0 to week 16. Fat intake was significantly higher in the probiotics group than in the placebo group at week 0 (*p* < 0.05). This difference was considered small and judged to be acceptable for conducting the study by the principal investigator. The investigators confirmed that the participants did not change their dietary habits before and after this study.

### 3.3. Effects on Body Composition and Blood Parameters

The measurement data are shown in Table 3. Due to unsuitable CT images, CT data were missing for five participants (placebo group; *n* = 3, probiotics group; *n* = 2). The probiotics group showed a significant reduction in their visceral fat area and total fat area compared to those in the placebo group (*p* < 0.05). There were no significant group differences in the subcutaneous fat area. The placebo group showed significant increases in body fat mass, body fat percentage, body weight, and BMI from baseline to week 16 (*p* < 0.05). In contrast, no significant increases were observed in the probiotics group. In particular, the increase in BMI was significantly suppressed in the probiotics group compared to the placebo group (*p* < 0.05). The probiotics group also had significantly decreased serum TG levels from baseline to week 16, and there was a significant group difference from the placebo group (*p* < 0.05). No significant differences were observed in cholesterol levels or markers related to blood glucose, liver function, and inflammation.

We also investigated the correlations between the reduction in visceral fat area and age or BMI by calculating the correlation coefficient. In addition, we compared the reduction in visceral fat area in males and females in the probiotics group by a two-sample *t* test. The reduction in visceral fat was shown to be independent of age, BMI, or sex.

### 3.4. Safety Assessment

There were 51 adverse events observed in the participants’ diaries and in blood and urine tests during the study period. The common adverse events were common cold or COVID-19 (11 events), cavity (5 events), and fluctuations in triglyceride (5 events). There was no significant difference in the incidence of adverse events between the two groups. All adverse events were judged to be non-serious by the principal investigator. None of the adverse events were related to the intake of test foods, and there were no side effects.

## 4. Discussion

In this study, 16 weeks of *B. longum* BB536 and *B. breve* MCC1274 intake significantly reduced the visceral fat area and total fat area in healthy normal and overweight adults compared to the placebo group. BMI and TG levels also showed significant differences between the groups. A previous study showed that 1 × 10^10^ CFU of *B. longum* BB536, 5 × 10^9^ CFU of *B. breve* MCC1274 and 250 mg of N-acetylglucosamine reduced the total fat area and BMI after 24 weeks of intervention [14]. Similarly, the present study showed the efficacy of these probiotics without any substrates on a wide range of obesity-related parameters. Overweight and obesity are known to occur regardless of age, sex, or race [1]. In addition, visceral fat has been suggested to be associated with the risk of diseases such as metabolic syndrome [16]. Therefore, prevention and improvement in fat accumulation are important for maintaining health. In this study, participants’ lifestyles were monitored with a diary, and they did not change their daily diet or exercise habits during the test period. Thus, 16 weeks of probiotic intake resulted in promising results for visceral fat area reduction. We also found that the reduction in visceral fat area was independent of age and sex, suggesting that healthy normal and overweight adults may benefit from probiotics.

It has been reported that there is seasonal variation in body fat levels, with an increase from summer to winter [17]. This study was conducted from summer to winter, and a significant increase was observed in the placebo group (Table 3). In the probiotics group, there was no significant increase in body fat from baseline, suggesting that the intake of *B. longum* BB536 and *B. breve* MCC1274 suppressed the increase in body fat level. On the other hand, the significant differences between groups in body fat level that were observed in previous studies with *B. breve* MCC1274 alone were not observed in this study [11,12,13]. The number of bacteria consumed were 2 × 10^10^ or 5 × 10^10^ CFU [11,12], and the method of body fat measurement was dual-energy X-ray absorptiometry (DEXA) [13], so the test conditions of previous studies were different from those of this study. Therefore, we consider that the body fat results in the present study may not be inconsistent with those of previous studies.

The intake of *B. longum* BB536 and *B. breve* MCC1274 resulted in a significant reduction in abdominal fat level. It is known that obesity is associated with impaired intestinal barrier function, and proinflammatory agents such as LPS can translocate into the blood [18]. Inflammation is reported to induce excess fat accumulation or insulin resistance [18,19,20]. A previous study showed that *B. breve* MCC1274 could promote *Claudin-1* gene expression and enhance the intestinal barrier function in vitro [9]. Therefore, it is possible that *B. breve* MCC1274 contributed to the fat level reduction via the suppression of inflammation by enhancing the intestinal barrier function. A previous clinical study of *B. breve* MCC1274 showed a trend towards a decrease in the levels of hCRP, an inflammatory marker [11]. In the present study, the hCRP levels at baseline and week 16 were low and in the healthy range. We hypothesize that the inflammation was too low to assess the effect of probiotics, because the participants were all healthy. The previous study included some participants who were being treated for diabetes, hypertension, or hyperlipidemia. Since chronic inflammation can occur in these diseases [21,22,23], it is assumed that the anti-inflammatory effect of probiotics is apparent when inflammation occurs.

Recently, the beneficial effects of postbiotics such as bacterial metabolites have been reported [24]. *B. longum* BB536 and *B. breve* MCC1274 are known to produce acetic acid, which is a short-chain fatty acid [9,25]. Short-chain fatty acids are reported to promote the expression of *GPR41* in the sympathetic nervous system and *GPR43* in adipose tissue [26,27]. These genes are reported to enhance energy consumption or suppress fat accumulation. It is assumed that the production of acetic acid might promote the expression of these genes and contribute to the reduction in visceral fat area. *B. longum* BB536 and *B. breve* MCC1274 also have a high capacity for indole-3-lactate (ILA) production [28]. ILA activates the aryl hydrocarbon receptor (AhR), and AhR activation is implicated in the enhancement of the intestinal barrier function and reduction in inflammation [29,30,31]. Furthermore, *B. breve* MCC1274 has a high capacity for conjugated linoleic acid (CLA) production; CLA is a type of unsaturated fatty acid that is produced from linoleic acid and has been reported to be a metabolic influencer, including antiobesity and antidiabetes activities [32]. For example, CLA administration has been reported to suppress body weight gain in ob/ob mice, decrease blood IL-1β levels, and increase the expression of *ZO-1*, an intestinal barrier factor [33]. Therefore, the postbiotics produced by *B. longum* BB536 and *B. breve* MCC1274 could have produced a fat-reducing effect.

In addition, this study showed a significant decrease in blood TG levels in the probiotics group. The administration of fermented milk with *B. longum* BB536 suppressed the elevation of blood TG levels compared to those with fermented milk without *B. longum* BB536 in vivo [8]. *B. longum* APC1472, which is the same species as *B. longum* BB536, inhibited weight gain and fat accumulation induced by a high-fat diet in vivo [34]. Blood corticosterone levels were also significantly lower in the APC1472 group than in the control group. Corticosterone (cortisol in humans) is a hormone induced by chronic stress and is suggested to be associated with visceral fat accumulation [35]. The lower level of corticosterone was considered to be one of the mechanisms of the beneficial effect in the APC1472 group. It was speculated that a similar mechanism may be mediated by *B. longum* BB536, which belongs to the same bacterial species. *B. breve* MCC1274 was also reported to inhibit weight gain and fat accumulation in vivo [10]. In the MCC1274 group, increased expression of lipid metabolism genes was observed. Therefore, it is assumed that *B. longum* BB536 or *B. breve* MCC1274 decreased TG levels through hormonal regulation and lipid metabolism enhancement. TG levels are known to correlate with the visceral fat area in non-diabetic obese adults [36], so the reduction in TG and visceral fat levels could be reasonable. In summary, *B. longum* BB536 and *B. breve* MCC1274 appeared to have beneficial effects on body composition and serum lipid levels through anti-inflammatory activity, postbiotic production, and lipid metabolism.

No side effects were observed after 16 weeks of consumption of the test food, confirming its safety. *B. longum* BB536 and *B. breve* MCC1274 have been shown to be safe in multiple clinical studies and have received safety certifications as being generally recognized as safe (GRAS) (BB536; GRAS notice No. GRN 268, MCC1274; GRAS notice No. GRN 1002). In this study, the combination of these probiotics was confirmed to be safe as well. There are some limitations in this study. The participants were only healthy normal and overweight adults, so the effects on children or patients with obesity or diabetes are not clear. Additionally, an effect on abdominal fat was observed at 16 weeks, but the effect at shorter periods is unknown. Therefore, further investigation is needed to clarify the effects of *B. longum* BB536 and *B. breve* MCC1274.

## 5. Conclusions

This study showed that *B. longum* BB536 and *B. breve* MCC1274 may reduce visceral fat and total fat levels in healthy normal and overweight adults compared to the placebo group. In addition, BMI and TG levels also showed significant differences between the groups. Participants did not change their daily diet or exercise habits, so 16 weeks of probiotic intake showed a positive effect on visceral fat reduction.

## Figures and Tables

**Table 1:** Baseline characteristics of the participants in the PPS population.

	Placebo (*n* = 47)	Probiotics (*n* = 47)	*p* Value
	Mean ± SD	Mean ± SD	
Age	50.11 ± 7.21	50.43 ± 7.59	0.835 ^b^
Sex (Male/Female)	33/14	33/14	1.000 ^c^
Total fat area (cm^2^) ^a^	315.68 ± 68.52	316.61 ± 58.16	0.953 ^b^
Visceral fat area (cm^2^) ^a^	112.77 ± 36.61	115.32 ± 35.91	0.741 ^b^
Subcutaneous fat area (cm^2^) ^a^	202.91 ± 37.80	201.28 ± 35.65	0.904 ^b^
Body fat mass (kg)	22.45 ± 4.68	22.55 ± 5.07	0.928 ^b^
Body fat percentage (%)	30.65 ± 6.83	30.83 ± 7.22	0.903 ^b^
Body weight (kg)	73.74 ± 7.69	73.55 ± 7.51	0.900 ^b^
BMI (kg/m^2^)	26.02 ± 1.87	26.20 ± 1.77	0.643 ^b^

^a^: Three participants in the placebo group and two participants in the probiotics group were excluded due to missing CT data. ^b^: *p* values determined by a two-sample *t* test. ^c^: *p* values determined by Fisher’s exact test.

**Table 2:** Nutrition intake during the intervention.

	Group	Week 0	Week 16	*p* Value (vs. Week 0) ^b^
		Mean ± SD	Mean ± SD	
Energy (kcal/day)	Placebo (*n* = 47)	1761.1 ± 385.6	1799.7 ± 456.4	0.527
	Probiotics (*n* = 47)	1851.9 ± 516.7	1871.6 ± 362.9	0.767
	*p* value ^a^	0.337	0.400	
Protein (g/day)	Placebo (*n* = 47)	64.0 ± 16.0	66.4 ± 18.7	0.344
	Probiotics (*n* = 47)	67.0 ± 18.6	70.2 ± 14.2	0.154
	*p* value ^a^	0.399	0.269	
Fat (g/day)	Placebo (*n* = 47)	57.4 ± 17.7	60.0 ± 21.8	0.367
	Probiotics (*n* = 47)	67.5 ± 23.0	67.5 ± 20.1	0.987
	*p* value ^a^	0.018	0.088	
Carbohydrates (g/day)	Placebo (*n* = 47)	222.0 ± 56.9	236.2 ± 57.3	0.064
	Probiotics (*n* = 47)	226.0 ± 69.0	232.6 ± 48.4	0.467
	*p* value ^a^	0.758	0.741	
Fiber (g/day)	Placebo (*n* = 47)	10.6 ± 3.5	11.4 ± 3.7	0.173
	Probiotics (*n* = 47)	10.7 ± 4.5	11.3 ± 3.0	0.410
	*p* value ^a^	0.869	0.907	

^a^: *p* values determined by a two-sample *t* test. ^b^: *p* values determined by a paired *t* test.

**Table 3:** Measurement of body composition and blood parameters.

	Group	Baseline	Week 16	*p* Value (vs. Baseline) ^c^
		Mean ± SEM	Mean ± SEM	
CT measurement				
Total fat area (cm^2^) ^a^	Placebo (*n* = 44)	315.68 ± 11.71	320.95 ± 11.04	0.115
	Probiotics (*n* = 45)	316.61 ± 10.43	312.42 ± 10.87	0.233
	*p* value ^b^	0.049		
Visceral fat area (cm^2^) ^a^	Placebo (*n* = 44)	112.77 ± 5.52	115.69 ± 5.70	0.199
	Probiotics (*n* = 45)	115.32 ± 5.35	111.50 ± 5.31	0.068
	*p* value ^b^	0.031		
Subcutaneous fat area (cm^2^) ^a^	Placebo (*n* = 44)	202.91 ± 10.33	205.26 ± 10.09	0.280
	Probiotics (*n* = 45)	201.28 ± 8.67	200.91 ± 9.17	0.864
	*p* value ^b^	0.373		
Bioelectrical impedance analysis				
Body fat mass (kg)	Placebo (*n* = 47)	22.45 ± 0.68	23.32 ± 0.64	<0.001
	Probiotics (*n* = 47)	22.54 ± 0.74	22.86 ± 0.79	0.272
	*p* value ^b^	0.107		
Body fat percentage (%)	Placebo (*n* = 47)	30.64 ± 1.00	31.45 ± 0.92	<0.001
	Probiotics (*n* = 47)	30.83 ± 1.05	31.10 ± 1.08	0.401
	*p* value ^b^	0.158		
Other parameters				
Body weight (kg)	Placebo (*n* = 47)	73.74 ± 1.12	74.64 ± 1.14	<0.001
	Probiotics (*n* = 47)	73.55 ± 1.10	73.82 ± 1.13	0.244
	*p* value ^b^	0.056		
BMI (kg/m^2^)	Placebo (*n* = 47)	26.02 ± 0.27	26.35 ± 0.27	<0.001
	Probiotics (*n* = 47)	26.20 ± 0.26	26.28 ± 0.27	0.319
	*p* value ^b^	0.046		
Triglycerides (mg/dL)	Placebo (*n* = 47)	120.49 ± 7.84	127.57 ± 10.44	0.342
	Probiotics (*n* = 47)	130.87 ± 11.78	108.98 ± 7.29	0.028
	*p* value ^b^	0.021		

^a^: Three participants in the placebo group and two participants in the probiotics group were excluded due to missing CT data. ^b^: *p* values determined by ANCOVA adjusted for the baseline. ^c^: *p* values determined by a paired *t* test.

## Data Availability

The data presented in this study can be found in this published article.
